# Intraoperative blood loss as indicated by haemoglobin trend is a predictor for the development of postoperative spinal implant infection—a matched-pair analysis

**DOI:** 10.1186/s13018-021-02537-9

**Published:** 2021-06-18

**Authors:** Friederike Schömig, Justus Bürger, Zhouyang Hu, Axel Pruß, Edda Klotz, Matthias Pumberger, Christian Hipfl

**Affiliations:** 1 Center for Musculoskeletal Surgery, Charité – University Medicine Berlin, Charitéplatz 1, 10117 Berlin, Germany; 2 Institute of Transfusion Medicine, Charité – University Medicine Berlin, Charitéplatz 1, 10117 Berlin, Germany; 3 Department of Anesthesiology and Intensive Care Medicine, Charité – University Medicine Berlin, Charitéplatz 1, 10117 Berlin, Germany

**Keywords:** Spine, Anemia, Infection, Diagnosis, Orthopaedic surgery

## Abstract

**Background:**

With a reported rate of 0.7–20%, postoperative spinal implant infection (PSII) is one of the most common complications after spine surgery. While in arthroplasty both haematoma formation and perioperative blood loss have been identified as risk factors for developing periprosthetic joint infections and preoperative anaemia has been associated with increased complication rates, literature on the aetiology of PSII remains limited.

**Methods:**

We performed a matched-pair analysis of perioperative haemoglobin (Hb) and haematocrit (Hct) levels in aseptic and septic spine revision surgeries. 317 patients were included, 94 of which were classified as septic according to previously defined criteria. Patients were matched according to age, body mass index, diabetes, American Society of Anesthesiologists score and smoking habits. Descriptive summaries for septic and aseptic groups were analysed using Pearson chi-squared for categorical or Student t test for continuous variables.

**Results:**

Fifty patients were matched and did not differ significantly in their reason for revision, mean length of hospital stay, blood transfusion, operating time, or number of levels operated on. While there was no significant difference in preoperative Hb or Hct levels, the mean difference between pre- and postoperative Hb was higher in the septic group (3.45 ± 1.25 vs. 2.82 ± 1.48 g/dL, *p* = 0.034).

**Conclusions:**

We therefore show that the intraoperative Hb-trend is a predictor for the development of PSII independent of the amount of blood transfusions, operation time, number of spinal levels operated on and hospital length of stay, which is why strategies to reduce intraoperative blood loss in spine surgery need to be further studied.

## Introduction

Recently, the number of spine surgeries performed worldwide has been steadily increasing [1, 2]. Even though improvements in surgical technique and postoperative care have been made, postoperative spinal implant infections (PSIIs) still occur in 0.7–20% of adult instrumented spine surgeries and are associated with higher patient morbidity and mortality, extended hospitalisation, worse long-term outcomes and increased health care costs [3, 4]. Currently, literature on the etiology, diagnosis and treatment of PSII remains limited in contrast to that on periprosthetic joint infections (PJIs). However, identification of risk factors and their impact on the development of PSII is crucial in order to prevent infection.

Anaemia has been shown to be a common comorbidity in patients undergoing surgery and to be increasing with advancing age. Multiple studies have examined the effects of preoperative anaemia on the development of postoperative complications such as mortality, length of hospital stay and infection in orthopaedic surgery in general. In the field of arthroplasty, preoperative anaemia was found to increase complication rates including the development of PJI [5–7].

Furthermore, both haematoma formation and perioperative blood loss have been previously identified as risk factors for developing PJI [8, 9]. One of the emerging measures to decrease their incidence is the application of tranexamic acid (TXA), which is a synthetic lysine analogue binding to plasminogen and thereby inhibiting the formation of plasmin. It has been shown to both safely and effectively reduce perioperative blood loss and transfusion requirements in different fields of medicine including trauma and surgery and specifically in total joint arthroplasty [10–14].

While the effects of anaemia and blood loss have been extensively studied in arthroplasty, to our knowledge no previous studies have examined the association of perioperative anaemia and the development of PSII. Thus, we performed a strict matched-pair analysis in aseptic and septic spine revision surgeries.

## Materials and methods

### Patients

The study was approved by the institutional ethics committee (EA2/128/19). We retrospectively included patients who underwent revision surgery after instrumented spine surgery at our institution between May 2015 and September 2020 with one or more microbiological and/or histopathological examinations of peri-implant tissue specimen and/or spinal implants submitted for sonication. Exclusion criteria were missing tissue and/or sonication samples.

Perioperative laboratory values for the index surgery, i.e. haemoglobin (Hb) and haematocrit (Hct), and clinical data including the number of peri- and intraoperative blood transfusions, body-mass-index (BMI) and comorbidities, i.e. diabetes, smoking habits, American Society of Anesthesiologists (ASA) scores, length of hospital stay, spinal levels operated on and operation time were retrieved from electronic medical reports and patient charts. Anaemia was defined as Hb < 13 g/dL for men and Hb < 12 g/dL for women. Hct cutoffs were defined as < 0.37 L/L for men and < 0.35 L/L for women.

### Patient blood management

Intraoperative blood loss was monitored and treated according to a standardised protocol. Monitoring of blood loss included measurement of blood volume collected by suction as well as frequent blood gas analyses with measurement of Hb and Hct. Decisions regarding blood transfusion were individualised according to the guidelines by the German Medical Association, which recommend a restrictive transfusion strategy tolerating Hb levels of 7–8 g/dL in stable patients while in patients with preexisting cardiovascular disease, a strict transfusion strategy with transfusion at Hb levels of 8 g/dL or less is suggested [15]. Thus, during the study period, in haemodynamically stable patients, a threshold of Hb ≤ 7 g/dL was maintained. Patients with a history of cardiovascular diseases were transfused at Hb ≤ 8 g/dL.

### Definition of PSII

As previously described, patients were classified as septic if at least one of the following criteria was met [16]: (1) acute inflammation or peri-implant membrane types II or III as defined by Krenn et al. in histopathological examination of peri-implant tissue [17]; (2) positive tissue culture with detection of low-virulent microorganisms in at least two specimen, detection of low-virulent microorganisms in one specimen confirmed by the same microbial growth in sonicate fluid culture, or detection of high-virulent microorganisms in at least one specimen; or (3) positive sonication culture with detection of > 50 colony-forming units (CFU) of low virulent bacteria, < 50 CFU of low-virulent bacteria with the same microbial growth in peri-implant tissue culture or detection of high virulent bacteria of any amount. Low-virulent microorganisms included coagulase-negative Staphylococci, Propionibacteria, *Bacillus* spp., *Fingeoldia* magna and *Enterococcus* spp. *Staphylococcus aureus*, *Streptococcus* spp. and Enterobacteria were classified as high-virulent microorganisms.

### Statistical analysis

Patients were matched according to age, BMI, diabetes, ASA score and smoking habits. Descriptive summaries for the septic and aseptic group were analysed using Pearson chi-squared for categorical or Student t test for continuous variables. For all tests, a *p* value of < 0.05 was considered significant. SPSS version 27 (SPSS Inc., Chicago, Illinois) was used for statistical analysis.

## Results

We identified 317 patients, 40 of which were excluded due to missing microbiological samples. In 94 patients, PSII was diagnosed using the criteria outlined. Fifty of these septic patients were matched with 50 aseptic patients. The mean age of the study group was 65 ± 21 years. Forty-four female patients were included (44%). The most common reasons for revision surgery were implant failure (53%) and adjacent segment degeneration (26%). Demographics as well as the clinical and laboratory data are shown in Table 1.

**Table 1:** Demographic, clinical and laboratory variables in both the septic and aseptic group. *P* values were calculated using Pearson chi-squared or Student’s t test. BMI, body mass index; ASA, American Society of Anesthesiologists

Variables	Septic group	Aseptic group	***p***-value
Age (years)	65 ± 21	65 ± 21	1.000
Gender (male to female)	28:22	28:22	1.000
BMI (kg/m^2^)	26.2 ± 5.6	29.0 ± 7.0	0.058
Diabetes, n (%)	8 (16.0)	14 (28.0)	0.114
Smoking, n (%)	6 (12.0)	6 (12.0)	1.000
ASA grade	2.4 ± 0.6	2.5 ± 0.6	0.471
Operation time (min)	171.3 ± 86.4	210.1 ± 123.0	0.062
Spinal levels operated on (n)	5.7 ± 4.2	5.5 ± 3.5	0.755
Perioperative blood transfusion (mL)	220.0 ± 446.0	175.1 ± 309.4	0.561
Hospital length of stay (days)	17.1 ± 19.2	12.5 ± 7.9	0.104
Reasons for revision, n (%)			0.183
Implant failure	22 (44.0)	31(62.0)	
Adjacent segment degeneration	13 (26.0)	13 (26.0)	
Wound disorders	10 (20.0)	2 (4.0)	
Infection	3 (6.0)	0 (0.0)	
Others	2 (4.0)	4 (8.0)	

Between septic and aseptic groups, there were no differences in age, gender, BMI, number of patients with diabetes, smoking or ASA grade. The mean time interval between index and revision surgery was 28.0 ± 25.6 months in the aseptic group and 18.7 ± 25.7 months in the septic group (*p* = 0.012). The mean length of hospital stay for the index surgery was 12.5 ± 7.9 days in the aseptic group and 17.1 ± 19.2 days in the septic group (*p* = 0.104). In the aseptic group, 5.5 ± 3.5 spinal levels were operated on, in the septic group 5.7 ± 4.2 levels (*p* = 0.755). The operating time for the index surgery was 210.1 ± 123.0 minutes in the aseptic group and 171.3 ± 86.4 minutes in the septic group (*p* = 0.062). The mean amount of perioperatively transfused erythrocyte concentrates was 175.1 ± 309.4 mL in the aseptic and 220.0 ± 446.0 mL in the septic group (*p* = 0.561), intraoperatively 13.5 mL ± 66.0 mL was transfused in the aseptic and 64.7 ± 174.9 mL was transfused in the septic group (*p* = 0.057).

Table 2 shows all retrieved laboratory data. The mean preoperative Hb was 13.15 ± 1.53 g/dl in the aseptic group and 13.68 ± 1.82 g/dl in the septic (*p* = 0.079), and mean preoperative Hct was 0.39 ± 0.04 in the aseptic and 0.40 ± 0.05 L/L in the septic group (*p* = 0.392). As shown in Table 3, in the aseptic group, 15 patients (30%) showed reduced Hb preoperatively while in the septic group, eleven patients (22%) did (*p* = 0.247). Preoperative Hct was reduced in nine patients (18%) in the aseptic group with ten patients (20%) in the septic group showing reduced Hct (*p* = 0.500). The mean difference between pre- and postoperative Hb was 2.82 ± 1.48 g/dL in the aseptic group and 3.45 ± 1.25 g/dL in the septic (*p* = 0.034). The mean difference between pre- and postoperative Hct was 0.08 ± 0.04 L/L in the aseptic group and 0.10 ± 0.04 L/L in the septic group (*p* = 0.055).

**Table 2:** Analysis of perioperative Hb and Hct values of the index surgery. Δ indicates preoperative minus postoperative value. *p* values were calculated using Student’s t test. *Significant differences. Hb, haemoglobin; Hct, haematocrit; SD, standard deviation

	Septic group (*n* = 50) Mean ± SD	Aseptic group (*n* = 50) Mean ± SD	***p*** value
**Haemoglobin**			
Preoperative Hb	13.68 ± 1.82	13.15 ± 1.53	0.079
Postoperative Hb	10.23 ± 1.65	10.33 ± 1.68	0.771
HBΔ	3.45 ± 1.25	2.82 ± 1.48	0.034*
**Haematocrit**			
Preoperative Hct	0.40 ± 0.05	0.39 ± 0.04	0.392
Postoperative Hct	0.30 ± 0.04	0.31 ± 0.05	0.383
HCTΔ	0.10 ± 0.04	0.08 ± 0.04	0.055

**Table 3:** Total numbers and frequencies of lowered Hb and Hct values pre- and postoperatively. Low Hb was defined as < 13.0 g/dl in men and 12.0 g/dl in women, low Hct as < 0.37 L/L in men and 0.35 L/L in women. *p* values were calculated using Pearson chi-squared test. Hb, haemoglobin; Hct, haematocrit

	Septic group (*n* = 50)	Aseptic group (*n* = 50)	***p***-value
**Preoperative**			
Hb, n (%)	11 (22.0)	15 (30.0)	0.247
Hct, n (%)	10 (20.0)	9 (18.0)	0.500
**Postoperative**			
Hb, n (%)	45 (90.0)	42 (84.0)	0.277
Hct, n (%)	45 (90.0)	41 (82.0)	0.194

## Discussion

This is the first study investigating the correlation between perioperative anaemia or Hb- and Hct-trend and the occurrence of PSII. While the results of our matched-pair analysis do not show a higher risk of PSII in patients with preoperative anemia, we were able to identify a negative Hb-trend during surgery as a predictor of developing an implant-associated infection. This result was independent of the amount of blood transfusions received intra- or perioperatively, the number of spinal levels operated on, operation time and hospital length of stay, all three of which did not show significant differences between the septic and aseptic groups.

As the literature on the treatment and prevention of PSII remains limited, strategies are often adapted from what has been found in PJI. In the attempt to reduce rates of PJI, modifiable perioperative risk factors were identified by large database analyses. In 2015, it was shown that preoperative anaemia is the most important modifiable predictor of major complications after total joint arthroplasty, including PJI. This is even more relevant considering that the prevalence of anaemia was 20% in the study population [18]. Even though our results do not show a relationship between preoperative anaemia and the development of PSII, we also show a high prevalence of preoperative anaemia of 20% in the septic and 30% in the aseptic group. While it may not be a predictor of PSII, preoperative anaemia may still be the cause of other major complications, which remains to be investigated.

Due to the abovementioned relationship between preoperative anaemia and the development of PJI, more recently, blood conservation strategies have gained attention in the field of arthroplasty. Here, multiple studies have shown that reducing intraoperative blood loss by perioperative application of TXA reduces the risk for PJI after both primary and revision total joint arthroplasty [19, 20]. While its effectiveness in reducing blood loss and thereby transfusion rates has been proven by several studies, its exact role in reducing this risk of infection has yet to be studied in depth. Some of the proposed mechanisms of action include the reduction of transfusions needed and of haematoma formation and thereby reduction of growth medium for pathogens [19]. This is further supported by the fact that high rates of haematoma and blood transfusion are known risk factors for developing PJI [8, 9]. However, a study reporting lower infection rates after TXA application in cardiac surgery found this effect to be independent of blood loss and rather to be an effect on plasmin-mediated immune-modulating pathways [21]. Our results show that in spinal surgery, higher intraoperative blood loss—indicated by higher intraoperative loss of Hb—increases the risk of developing PSII. This supports the more recent notion in arthroplasty that reducing intraoperative blood loss reduces the risk for PJI. Therefore, in spine surgery, a reduction of intraoperative blood loss by measures such as the perioperative application of TXA seems to be reasonable as well, which is why prospective studies would certainly be of value in this regard.

Our study has several limitations. As it was conducted as a retrospective study, inherent limitations and bias were present and due to a rather small sample size, the statistical power was limited. However, to our knowledge, this still is the largest matched-pair analysis of PSII. Not all patients with suspected PSII could be included as in several cases histopathological and microbiological samples had not been collected intraoperatively. This might have caused selection bias as samples may have been collected preferentially in patients with macroscopical signs of infection. By performing a matched-pair analysis, we did however reduce this bias to a minimum. Furthermore, to date there is no accepted definition for the diagnosis of PSII, which is why the interpretation of microbiological results of peri-implant tissue cultures and sonication differs among institutions. However, in this study, strict criteria were used for defining septic revisions, which is why we are confident that our results are robust.

## Conclusions

In conclusion, while our results do not confirm preoperative anaemia as a risk factor for the development of PSII, we were able to show that intraoperative Hb trend as an indicator of intraoperative blood loss is a predictor of an increased risk for the development of PSII independent of the amount of blood transfusions, operation time, number of spinal levels operated on and hospital length of stay. Therefore, strategies to reduce intraoperative blood loss should be further studied in order to prevent PSII as a major complication in instrumented spine surgery.

## Data Availability

The datasets generated and/or analysed during the current study are not publicly available due to patient privacy but are available from the corresponding author on reasonable request.

## Abbreviations

ASA: American Society of Anesthesiologists
BMI: Body mass index
CFU: Colony-forming units
Hb: Haemoglobin
Hct: Haematocrit
PJI: Periprosthetic joint infection
PSII: Postoperative spinal implant infection
SD: Standard deviation
TXA: Tranexamic acid
